# Targeting the Tumor Microenvironment: An Unexplored Strategy for Mutant KRAS Tumors

**DOI:** 10.3390/cancers11122010

**Published:** 2019-12-13

**Authors:** Patrícia Dias Carvalho, Ana Luísa Machado, Flávia Martins, Raquel Seruca, Sérgia Velho

**Affiliations:** 1 i3S – Instituto de Investigação e Inovação em Saúde, Epithelial Interactions in Cancer group, Rua Alfredo Allen, 4200-135 Porto, Portugal; pcarvalho@ipatimup.pt (P.D.C.); almachado@i3s.up.pt (A.L.M.); flaviam@ipatimup.pt (F.M.); rseruca@ipatimup.pt (R.S.); 2 IPATIMUP – Institute of Molecular Pathology and Immunology of the University of Porto, Rua Júlio Amaral de Carvalho 45, 4200-135 Porto, Portugal; 3 ICBAS - Institute of Biomedical Sciences Abel Salazar (ICBAS), University of Porto, R. Jorge de Viterbo Ferreira 228, 4050-313 Porto, Portugal; 4 INEB – Institute of Biomedical Engineering, Tumor and Microenvironment group, Rua Alfredo Allen, 4200-135 Porto, Portugal; 5 Ciências Químicas e das Biomoléculas, Centro de Investigação em Saúde e Ambiente, Escola Superior de Saúde do Porto, Instituto Politécnico do Porto, 4200-072, Porto, Portugal; 6 Department of Pathology, FMUP – Faculty of Medicine of the University of Porto, 4200-319 Porto, Portugal

**Keywords:** KRAS, tumor microenvironment, cancer therapy, immunotherapy, lung cancer, pancreatic cancer, colorectal cancer

## Abstract

Current evidence strongly suggests that cancer cells depend on the microenvironment in order to thrive. In fact, signals from the surrounding tumor microenvironment are crucial for cancer cells´ aggressiveness, altering their expression profile and favoring their metastatic potential. As such, targeting the tumor microenvironment to impair cancer progression became an attractive therapeutic option. Interestingly, it has been shown that oncogenic KRAS signaling promotes a pro-tumorigenic microenvironment, and the associated crosstalk alters the expression profile of cancer cells. These findings award KRAS a key role in controlling the interactions between cancer cells and the microenvironment, granting cancer a poor prognosis. Given the lack of effective approaches to target KRAS itself or its downstream effectors in the clinic, exploring such interactions may open new perspectives on possible therapeutic strategies to hinder mutant KRAS tumors. This review highlights those communications and their implications for the development of effective therapies or to provide insights regarding response to existing regimens.

## 1. Introduction

The high incidence of RAS isoforms—HRAS, NRAS, and KRAS—mutations in human cancer and its associated relevance in this disease has long been known and explored [1]. In fact, RAS is the most frequently mutated oncogene in human cancer, with mutation in the KRAS isoform the most commonly found. Briefly, KRAS proteins are small GTPases that function as signal transducers of extracellular stimuli from several different cell surface receptors (e.g., EGFR) to the interior of the cell. Mutations in this oncogene, either by inhibiting its ability to hydrolyze GTP or by promoting the rapid exchange of GDP for GTP, render the protein constitutively active [2]. This impacts several signaling pathways, such as RAF–MEK–ERK, PI3K–AKT–mTOR, and RALGDS–RAL, that control a myriad of essential cellular processes such as proliferation, growth, and survival, ultimately favoring cancer progression [3].

KRAS mutations are particularly frequent in pancreatic ductal adenocarcinoma (PDAC), colorectal (CRC), and nonsmall cell lung cancers (NSCLC) [2,3]. Importantly, these figure on the list of most deadly cancers worldwide, according to Globocan 2018, with a tendency to increase in incidence and mortality in the next years. The presence of a KRAS mutation is predictive of poor prognosis and therapy resistance [4,5,6], and indeed, mutant KRAS predicts resistance to anti-epidermal growth factor receptor (EGFR) treatments, leaving these patients with no efficient therapeutic options. Moreover, in some cases, different KRAS hotspot mutations have been associated with different sensitivities to commonly used therapeutic regimens [7,8,9], suggesting that it is important to analyze not only KRAS mutation status but also the specific mutation present. It is of major importance to unravel the KRAS-mediated effects that are underlying the different resistance mechanisms.

## 2. Current Approaches to Target Mutant KRAS Cells

**Figure 1:**
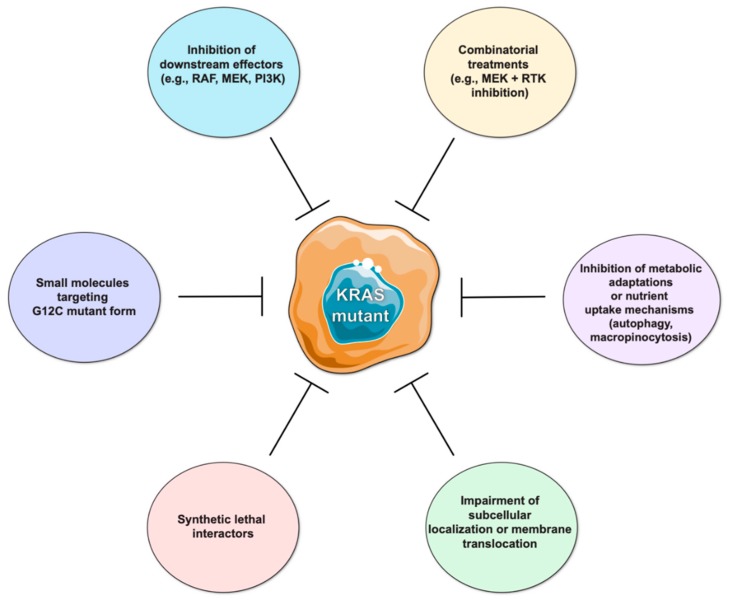
Strategies to target mutant KRAS cells. The lack of efficient therapies targeting mutant KRAS tumors represents an unmet clinical need. Several strategies have already been tested or are currently under development. Inhibitors of KRAS downstream effector molecules (e.g., RAF, MEK, PI3K) did not result in significant clinical benefit as standalone treatments, but their use in combination with receptor tyrosine kinase (RTK) inhibition has been shown to induce favorable antitumoral responses. The development of KRAS direct inhibitors represents a major breakthrough in the field, particularly of those targeting specific mutant forms, such as the G12C mutation, which are currently in clinical trials. Moreover, several other strategies under study aim to identify synthetic lethal interactors of KRAS, to impair KRAS post-translational modifications interfering with its subcellular localization, and to hamper the mechanisms used by mutant cells to obtain nutrients and energy.

KRAS mutations are well-known exclusion biomarkers for anti-EGFR targeted therapies, and decades of research have been dedicated to the development of approaches to impair tumors with activation of this oncogene (Figure 1).

Inhibition of single downstream effector molecules (e.g., RAF, MEK, or PI3K) did not produce major clinical benefits, where induction of compensatory mechanisms that reactivate the pathway or even activation of alternative KRAS signaling effectors may account for resistance mechanisms [10,11]. Nevertheless, since mutant KRAS cells seem to show increased dependence on receptor tyrosine kinase (RTK) signaling such as erythroblastic leukemia viral oncogene (ERBB) family, hepatocyte growth factor receptor (MET) and insulin growth factor receptor (IGFR), combined inhibition of specific signaling effectors and RTK (e.g., MEK and IGFR inhibition, or MEK and pan-ERBB inhibitors) have shown therapeutic potential [12,13,14].

Although pursued for many years without success, the development of small molecules targeting KRAS directly recently yielded promising results both in preclinical and clinical studies. In particular, KRAS G12C mutant-specific inhibitory molecules revealed an encouraging antitumor effect in preclinical models using cell lines of patient-derived samples as well as in phase I clinical trials with lung and CRC patients [15]. A major downside of targeting specifically G12C is that, despite accounting for about 50% of all KRAS mutations in lung cancer, it is not the most frequent amino acid change in CRC and pancreatic cancers, representing approximately 15% and 2% of all KRAS mutations found in these tumors, respectively [2]. Ultimately though, the possibility of targeting KRAS G12C reignited the hope on suppressing KRAS directly and on the development of novel inhibitors targeting other KRAS mutant forms.

Additional alternatives based upon the biochemical and biological processes associated with KRAS activation are being investigated to impair its oncogenic activity. These encompass targeting post-translational modifications to interfere with KRAS subcellular localization and translocation to the membrane, hindering the metabolic adaptations and scavenging pathways (e.g., authophagy and micropinocytosis) used by mutant KRAS cells for energy production and nutrient uptake [10,16,17,18,19]. In this regard, farnesyltransferase inhibitors were tested in the past as a strategy to limit KRAS translocation to the plasma membrane by impairing prenylation of the cysteine in the CAAX motif, the initial step of KRAS post-translational modifications. However, this was not successful in the clinic given that KRAS can be alternatively prenylated by geranylgeranyl transferases [20]. As an alternative, inhibition of the enzyme prenyl-binding protein phosphodiesterase δ (PDEδ), that binds prenylated KRAS and facilitates trafficking from the cytoplasm to the plasma membrane via Golgi and recycling endosomes, was shown to decrease KRAS oncogenic activity [21,22]. Inhibition of KRAS-enhanced macropinocytosis represents another possibility to target mutant cancer cells by interfering with their ability to internalize extracellular fluids-containing nutrients. Although the molecular basis of KRAS-driven macropinocytosis is not yet clearly demonstrated, KRAS-dependent expression of Syndecan 1 at the cell surface was recently identified as a molecular mechanism regulating macropinocytosis and indicated as a putative therapeutic target [23]. Moreover, the high internalization of albumin through macropinocytosis can be explored as a drug delivery strategy. As reported by Liu H and colleagues [24], doxorubicin effects were 10x higher when the drug was combined with albumin in in vitro and in vivo models of mutant KRAS PDAC. Aberrant KRAS signaling is also associated with a glycolytic metabolic profile highlighting alternative strategies to harness KRAS-associated effects. Specifically, mutant KRAS increases the levels of Glucose transport 1 protein at the cell surface, leading also to an increased expression of several other molecules involved in glucose metabolism, such as hexokinase 1 and 2, phosphofructokinase 1, enolase 1, lactate dehydrogenase A, as well as molecules involved in other biosynthetic pathways (glucosamine-fructose-6-phosphate aminotransferase 1, ribulose-5-phosphate-3 epimerase, ribulose-5-phosphate isomerase, aspartate transaminase) [11,25]. In addition, the identification of synthetic lethal interactors of KRAS may provide useful targets for therapeutic intervention in KRAS-driven cancers [26,27,28,29]. To date, several functional genetic screens have identified KRAS synthetic lethal interactors with targetable potential, including kinases and other molecules involved in pathways associated with proliferation and apoptosis (extensively reviewed in [28]). Despite being promising, oncogenic KRAS codependencies may vary between tumor types, according to the genetic background of the cell lines used and the KRAS mutation, limiting the capacity to validate the findings amongst different studies. Moreover, studies using patient samples, cultured in 3D conditions and in the presence of tumor microenvironment (TME) components, may provide more realistic insights on the factors that cooperate with mutant KRAS [28,29].

Despite these potentially effective options to target KRAS mutant cancer cells, none of them have yet been translated into clinical practice.

## 3. Exploring the Crosstalk between the Cancer Cell and the Tumor Microenvironment to Target Mutant KRAS Cancers

Oncogenic mutations are on the genesis of the carcinogenic process; however, the development and progression of solid tumors also strongly depends on the communication and coevolution with the surrounding TME. A plethora of cellular (e.g., fibroblasts, immune cells, pericytes, and adipocytes) and noncellular (e.g., extracellular matrix, cytokines, and chemokines) components constitute a complex, spatial and temporally dynamic environment with intricate communication routes that cooperate to instigate tumor progression. These TME components participate in tumor outgrowth, metastatic dissemination, colonization of secondary organs, awakening of dormant micrometastases, and influence the response to therapy, being key players in the induction of resistance mechanisms [30,31,32]. As such, new therapeutic TME-directed strategies are being explored to target or re-educate the TME components. Moreover, combining TME-directed therapies with inhibitors of tumor cell-intrinsic molecular features is becoming an appealing therapeutic approach to surpass the effect of oncogenic events [33,34].

The clinical importance of KRAS-driven alterations is not only awarded to how this oncogene favors cancer cells’ autonomous mechanisms but also to how it alters the way cancer cells interact and influence the TME components [35]. Therefore, the quest for alternative and effective treatment options for patients harboring mutant KRAS tumors should also focus on the crosstalk between mutant KRAS cancer cells and the surrounding TME. Below, we discuss the latest discoveries on the communication between mutant KRAS cancer cells and their microenvironment, emphasizing those that hold clinical potential for the tumor types where KRAS mutations are frequent.

### 3.1. Targeting Mutant KRAS-Driven Effects that Shape the Cancer-Immune Cell Crosstalk

When talking about the immune microenvironment in clinical cancer research, the hot topic concerns the development of immunotherapeutic tools to enable an immune response against tumors [36]. Indeed, therapies targeting immune-checkpoint molecules (e.g., programmed cell death 1 (PD-1), programmed cell death ligand 1 (PD-L1), and cytotoxic T-lymphocyte-associated protein 4 (CTLA-4)) are currently used in clinical practice for the treatment of several cancers [37]. In the case of CRC, immunotherapy is approved for the treatment of mismatch repair (MMR) deficient metastatic tumors refractory to other lines of treatment, representing only ~2–4% of all CRC patients [38]. While MMR proficient patients do not benefit from anti-PD-L1 therapies, MMR deficient ones yield a better, though not complete, overall response rate, and impressive improvements of progression-free survival rates [38,39]. In NSCLC, the use of anti-PD-L1/PD-1 therapy is clinically approved [37], where PD-L1 positive patients have been showing great responses. Nevertheless, studies involving therapies targeting other immune checkpoint molecules in NSCLC, such as anti-CTLA-4 antibodies, have shown more disappointing results [40]. Importantly, the use of immunotherapy in pancreatic cancer has so far resulted in limited clinical success, and therefore it is not yet included in the clinical guidelines. The “cold” nature of these tumors, as well as the abundance and complexity of their TME, may explain the lack of effectiveness of conventional immunotherapy treatments. Thus, combinatorial strategies targeting the immune system (e.g., PD-L1) and molecular inductors of pancreatic TME complexity (e.g., colony stimulating factor receptor 1(CSFR1), chemokine C-X-C receptor 4 (CXCR4)) are in clinical trials, and it is expected that the reprograming of the TME will improve the benefit of classical immunotherapy treatment [41].

**Figure 2:**
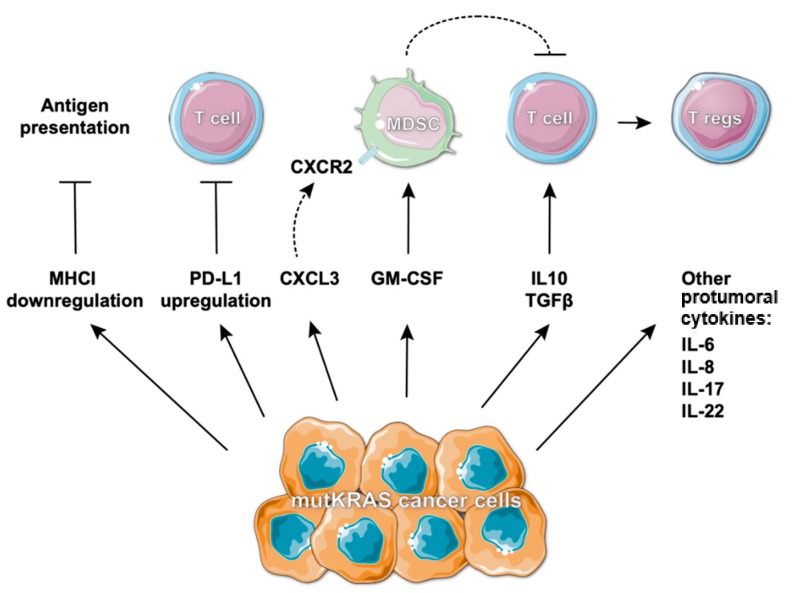
KRAS-induced immune-suppressive microenvironment. Mutant (mut)KRAS cells have been associated with decreased major histocompatibility class I (MHCI) expression, representing an impaired capacity to present antigens. Furthermore, the upregulation of programmed cell death ligand 1 (PD-L1) leads to immune evasion by inhibiting T-cell recognition. In these cells, the expression and secretion of several inflammatory cytokines is also recognized as being increased. Chemokine C-X-C ligand 3 (CXCL3) binds to its receptor chemokine C-X-C receptor 2 (CXCR2) on myeloid-derived suppressor cells (MDSCs) contributing to the maintenance and recruitment of these immune suppressive cells. In addition, granulocyte macrophage colony-stimulating factor (GM-CSF) is responsible for the accumulation of MDSCs in the tumor microenvironment. Moreover, the increased secretion of interleukin (IL)-10 and Transforming growth factor beta 1 (TGFβ1) induce the conversion of CD4+ CD25− T-cells into FOXP3+/CTLA4+/CD122+ T regulatory cells (Tregs) promoting immune suppression.

Independently of the obvious benefit in some cases, it is urgent to understand why some patients still do not respond to immunotherapy, to explore the underlying resistance mechanisms and to identify biomarkers that will allow better patient stratification and prediction of response. In the last few years, KRAS mutations were reported to influence and modulate the surrounding immune contexture. Intensive research has provided evidence that this oncogene impacts many aspects that directly or indirectly shape the immune repertoire within the TME [35] and ultimately the response to immunotherapies. Accordingly, Canon et al. recently demonstrated that treatment of KRAS G12C mutant tumors with a G12C-specific inhibitor induced a pronounced tumor regression and long-lasting responses when used alone or in combination with immunotherapy, by promoting a proinflammatory TME. Impressively, the growth of isogenic KRAS G12D tumors was also impaired in treated mice, indicative of immunological memory against shared antigens [42]. Meanwhile, in lung cancer, the majority of reports associate mutant KRAS with increased PD-L1 expression (Figure 2) [43,44,45,46,47] and improved clinical response to anti-PD-1 therapy [44,45]. In a KRAS mutant lung tumor model, the mechanism behind PD-L1 increased expression was already elucidated. Coelho et al. described a mechanism involving inhibition of tristetetrapolin (TTP), a negative regulator of PD-L1 expression, through KRAS-induced MEK signaling, which results in PD-L1 mRNA stabilization. In vivo, TTP activity restoration decreased PD-L1 expression, augmenting anti-tumor immunity [48]. In this case, PD-L1 increased expression was associated with immune evasion, indicating that KRAS mutant cancers could indeed benefit from treatment with anti-PD-L1 or anti-PD-1 immune checkpoint inhibitors. Interestingly, some works have already demonstrated that despite the lack of clinical relevance of MEK inhibitors when used as single agents, their combination with immunotherapies can result in increased clinical benefit [49,50,51,52]. These works claimed an effect of MEK inhibitors on the modulation of the immune-suppressive microenvironment that synergized with anti-PD-L1 or anti-CTLA-4 therapies to enhance treatment response [49,52]. Moreover, in NSCLC, KRAS activation is linked with the development of an inflammatory environment [53]. KRAS mutant NSCLCs can be classified in different subgroups according to co-occurring genetic events: the KRAS-LKB1 (KL group), the KRAS-TP53 (KP group), and the KRAS-CDKN2A/B (KC group). Although information regarding the immune composition of KC tumors is scarce, it is well established that KL tumors are poorly immune-infiltrated, express low levels of checkpoint inhibitor molecules, including PD-L1, and are thus resistant to anti-PD-1 therapies. In contrast, the KP group is characterized by dense T-cell infiltration, high expression of PD-L1, and increased response to anti-PD-1 therapy [54,55]. The molecular mechanism behind the immunotherapy resistance of KL tumors has been shown to involve the suppression of stimulator of interferon genes (STING) upon LKB1 loss. cGAS-STING signaling downregulation decreased the expression of type I interferon genes and chemokines that facilitate T-cell recruitment. In this context, reconstitution of either LKB1 or STING was sufficient to restore PD-L1 expression [56], opening a window to study this resistance mechanism in the search of therapeutic targets for KL tumors. Additionally, this subset of tumors is associated with the assembly of an immune-suppressive microenvironment through neutrophils accumulation, proinflammatory cytokine production, and reduced T-cell infiltration. Treatment with an interleukin (IL)-6 neutralizing antibody resulted in the modulation of the immune compartment towards a more anti-tumorigenic phenotype with functional T-cell infiltration and consequently improved mouse survival [57]. In addition to IL-6, many reports in KRAS-driven lung tumor models have been associating mutant KRAS with protumoral cytokines, such as IL-8, IL-17, and IL-22 (Figure 2), which participate in the modulation of the immune microenvironment towards a pro-tumorigenic phenotype. Many of the effects orchestrated by these cytokines seem to be mediated by the transcription factor Signal transducer and activator of transcription 3 (STAT3) and most importantly, blocking these cytokines resulted in inhibition of tumor progression with concurrent re-education of the immune microenvironment [58,59,60,61,62,63]. In addition, in a KRASG12D-driven lung cancer model, the IL-6-STAT3 axis was shown to be essential for the cachexia phenotype, demonstrating the importance of targeting this pathway in advanced tumors [64]. Konen and colleagues also described a mechanism involving STAT signaling underlying the resistance to anti-PD-1 therapy in the KP model. The authors showed that neurotrophic receptor tyrosine kinase 1 (NTRK1) is upregulated in tumors treated with PD-1 inhibitors. NTRK1 was found to regulate Janus kinase (JAK)/STAT signaling and consequently promote the expression of PD-L1 on tumor cells leading to CD8+ T-cell exhaustion in the microenvironment [65].

Aside from impacting the immune contexture of lung cancers, mutant KRAS contributes towards an immune-suppressive microenvironment in CRC, allowing the escape to immune recognition. In CRC, the presence of mutant KRAS is associated with downregulation of major histocompatibility class I (MHCI) molecules [66,67], which means that these cells have an impairment to present antigens (Figure 2) and are less prone to be detected by the immune system. A clinical trial (ClinicalTrials.gov Identifier: NCT03271047) testing the efficacy of combining the MEK inhibitor binimetinib with anti-PD-1 or anti-CTLA-4 agents (nivolumab/ipilimumab) is currently ongoing for cases of pretreated microsatellite stable metastatic CRC patients that harbor a RAS mutation. Results on this study will assess the clinical applicability of a combinatorial treatment in the setting of poorly immune infiltrated KRAS mutant CRC.

It has been also shown that mutant KRAS cells secrete IL-10 and transforming growth factor beta 1 (TGFβ1) (through activation of the MEK–ERK–AP-1 pathway) and consequently induce the conversion of CD4+ CD25- T-cells into FOXP3+/CTLA4+/CD122+ T regulatory cells (Tregs) (Figure 2). This was validated using a mouse model of lung cancer, supporting a role for mutant KRAS on the regulation of Tregs infiltration in different types of cancer [68]. Moreover, a recent study demonstrated that mutant KRAS CRC displayed low infiltration with CD4+ T-cells and CD4+/FOXP3+ Tregs but were highly infiltrated with myeloid-derived suppressor cells (MDSCs). MDSCs infiltration was shown to depend on mutant KRAS suppression of interferon responses via the regulation of interferon regulatory factor 2 (IRF2). Due to IRF2 downregulation, chemokine C-X-C ligand 3 (CXCL3) expression and secretion was significantly increased, which, upon binding to chemokine C-X-C receptor 2 (CXCR2) on MDSCs, promoted the development of an immune-suppressive microenvironment (Figure 2). Further, IRF2 expression or CXCR2 blockade in mutant KRAS-driven tumors overcomes resistance to anti-PD-1 therapy [69]. Accordingly, upregulation of CXCR2 has been also reported in PDAC KRAS-driven models, as well as the efficacy of blocking CXCR2 to restrain tumor growth [70]. These results strengthen the role of mutant KRAS in the induction of an immune-suppressive microenvironment, pinpointing the involvement of several cytokines and chemokines whose inhibition could be further investigated for the treatment of mutant KRAS CRC. For instance, TGFβ1 inhibition was shown to promote an anti-tumorigenic immune infiltration of immune-excluded CRC and restored sensitivity to PD-L1/PD-1 blockade [71].

Using mouse models of PDAC, the KRAS G12D mutation was shown to upregulate granulocyte macrophage colony-stimulating factor (GM-CSF), leading to accumulation of Gr1+CD11b+ MDSCs and creating a pro-tumorigenic immune microenvironment (Figure 2). In contrast, suppression of GM-CSF was sufficient to reduce the levels of Gr1+CD11b+ cells and to inhibit tumor growth [72,73]. Importantly, also in PDAC, targeting granulocytic MDSCs was shown to be beneficial as it leads to the accumulation of cytotoxic CD8+ T-cells and tumor cell apoptosis [74]. Considering that pancreatic cancers are recognized as “cold”/poorly immunogenic, a potentially effective approach to improve tumor-immune recognition may encompass the targeting of this subpopulation of cells. In accordance, in a KRAS G12D-driven mouse model of lung cancer, it was shown that Gr1+ cells not only favor tumor growth but also reduce the infiltration with T-cells, inhibiting anti-PD-1 efficacy. By acting on blood vessels, Gr1+ cells induced hypoxia, which in turn increased the expression of Snail via hypoxia inducible factor 1 subunit alpha (HIF-1α). Tumor cells expressing Snail augmented the secretion of CXCL2 on GR1+ cells leading to increased recruitment of these immune-suppressive cells [75]. In another KRAS-driven lung cancer mouse model, depletion of Gr-1^+^CD11b^+^ myeloid cells also suppressed tumor growth [60]. These results not only support the relevance of granulocytic suppressive cells on immune evasion but also reinforce the impact of chemokines belonging to the CXCL family on tumor progression. Targeting these KRAS-associated mechanisms can possibly sensitize tumors for immunotherapy.

Moreover, in addition to the effects in the orchestration of the immune microenvironment, treatment of KRAS-driven tumors with conventional therapy also proved to shape the immune landscape. For instance, when treated with chemotherapy, mutant KRAS PDAC cell lines were shown to secrete increased levels of inflammatory cytokines, such as GM-CSF, via activation of mitogen-activated protein kinase (MAPK) and factor nuclear kappa B (NF-kB) pathways. In turn, these inflammatory cytokines are able to direct monocyte differentiation towards MDSCs resulting in an unwanted side effect that can counteract therapy response. Neutralization of GM-CSF using monoclonal antibodies successfully blocked the observed effects, thus suggesting this as a potential candidate to adjuvant therapy [76].

In summary, mutant KRAS seems to influence the composition of the immune microenvironment through a multitude of mechanisms that are definitely context and tumor-type dependent. The study of these mechanisms is not only boosting our understanding on tumor-immune evasion and justifying the therapy resistance in some patients, but most importantly are highlighting possible therapeutic targets and stratification biomarkers. The development of combinatorial therapies directed against these KRAS-regulated mechanisms that instigate the formation of an immune-suppressive microenvironment can help improve patients’ response to already used immunotherapies.

### 3.2. Targeting the Crosstalk between Mutant KRAS Tumor Cells and Cancer-Associated Fibroblasts 

Cancer-associated fibroblasts (CAFs) play a key role in the acquisition and maintenance of several cancer hallmarks, being involved in cancer cell growth, migration, invasion, and metastization, immune regulation, and therapy response [77,78,79,80].

Using both a KRAS G12D genetically engineered mouse model of lung cancer and xenografting of CT26 mouse colon cancer cells, it was shown that genetic depletion or pharmacological targeting of fibroblast activation protein (FAP)-expressing cells decreased myofibroblasts content and impacted blood vessel density and extracellular matrix composition, ultimately inhibiting tumor cell proliferation/growth [81]. These results support a dependency of these epithelial tumors on the presence of FAP-expressing cells within the microenvironment and suggest that targeting these cells could function as an effective therapeutic strategy to treat these tumors. Nonetheless, other studies refer that depleting fibroblasts from tumors promotes disease progression. In PDAC, for instance, which is specifically characterized by a dense tumor stroma, suppression of the stromal desmoplasia results in acceleration rather than restraining of tumor progression. Genetic or pharmacological targeting of the Hedgehog (Hh) pathway, with consequent depletion of stromal cells, results in tumors with an undifferentiated morphology, increased proliferation and vascularization, and decreased mice survival [82,83]. Importantly, in PDAC cells, oncogenic KRAS was shown to be responsible for activation of the Hh pathway, being required for the maintenance of the surrounding active stroma [84,85]. Moreover, also in a PDAC mouse model, depletion of alpha smooth muscle actin (α-SMA)-expressing cells increased tumor invasion and reduced survival. Accordingly, a low α-SMA score in tumors from PDAC patients correlated with worse survival. Interestingly, myofibroblast-depleted tumors presented decreased infiltration with T- and B-cells along with an increase in CD4+ Foxp3+ Tregs and concurrent CTLA-4-increased expression. Despite the connection of myofibroblast depletion and decreased survival, in this context, anti-CTLA4 therapy led to increased overall survival [86], creating an opportunity for combinatorial therapeutic strategies.

Complete CAFs depletion from the TME may not be an ideal approach to impair tumor growth given their heterogeneity, plasticity, and diverse origins, which impact their function. More so, the lack of specific markers to distinguish between pro- and anti-tumorigenic CAF populations adds complexity to their usage in therapeutic strategies [80,87,88]. Regardless, oncogenic KRAS has been shown to stimulate a heterocellular signaling with stromal cells, that is essential to fuel tumor cell proliferation [89]. As so, targeting specific molecules involved in the crosstalk between fibroblasts and cancer cells may represent a reliable strategy deserving further understanding.

In KRAS-driven PDAC mouse models, CAFs were shown to overexpress CXCR2 ligands promoting migration, invasion, and angiogenesis, and in fact, blocking the CXCLs-CXCR2 axis resulted in tumor cell apoptosis, decreased vessel density, and modulation of the immune infiltrate. Specifically, this led to reduced MPO+ neutrophils, CD11+ Ly6G+ MDSCs and arginase-1 (Arg-1)-positive macrophages (M2-like), as well as increased inducible nitric oxide synthase (iNOS)-positive macrophages (M1-like), with consequent improvement in mouse survival [90,91]. Moreover, other reports described that CAFs expressing FAP mediate immune suppression by secretion of CXCL12. In this case, either FAP+ CAFs depletion or inhibition of the CXCL12 receptor-CXCR4 in combination with immune-checkpoint inhibitors resulted in a clear tumor reduction [92]. Given the number of studies showing the influence of KRAS on the CXCLs-CXCRs axis, further studies addressing this connection and its therapeutic potential can reveal promising approaches to target mutant KRAS cases that are refractory to immunotherapies. Moreover, in a PDAC mouse model, the tumor stroma was found to produce high levels of IL-6, whereas the tumor expressed high levels of PD-L1. Combination of IL-6 and PD-L1 blocking resulted in increased T-cell infiltration and reduced α-SMA+ CAFs [93]. Other authors have shown that the mechanism behind IL-6 production in fibroblasts is regulated by KRAS in a paracrine fashion. Oncogenic KRAS induces the expression of Sonic Hedgehog (SHH) in cancer cells, whilst SHH induces expression of the transcription factor GLI family zinc finger 1 (GLI1) in fibroblasts. GLI1 binds to IL-6 promoter and fibroblasts-secreted IL-6 regulates activation of STAT3 in cancer cells. In this model, loss of GLI1 impaired KRAS-induced pancreatic carcinogenesis [94], revealing a targetable KRAS-driven mechanism underlying the crosstalk between cancer cells and the pancreatic stromal compartment.

Targeting strategies encompassing CAFs-associated signaling and immunomodulation may therefore constitute an auspicious therapeutic approach.

## 4. Conclusions

Currently, mutant KRAS patients have neither efficient nor targeted therapies available and, therefore, there is an urgent need to understand KRAS-associated signals to unravel putative therapeutic targets. As it is reflected in the aforementioned works, there are therapeutic strategies that can potentially target KRAS mutant cancer cells directly, some of which are already being tested in clinical trials. Even if successful, only a fraction of KRAS mutant patients will be eligible, and the acquisition of resistance mechanisms is likely to occur [95]. Analysis of the TME of mutant KRAS tumors and the study of the molecular mechanisms involved in the crosstalk between mutant KRAS cancer cells and the TME has provided valuable information on the regulation of tumor initiation and progression. Moreover, it has been shown that the TME plays a considerable role in dictating the response to therapy and contributes to the acquisition of resistance mechanisms. As such, the influence exerted by the TME, as well as the crosstalk established between the TME components and the cancer cells, must be considered as they may reveal putative targets with clinical relevance and applicability, either alone or used in combinatorial treatment schemes. Notwithstanding the considerable advances related to the development of new anti-KRAS therapies, mutant KRAS patients will certainly benefit from a better understanding of the KRAS-orchestrated mechanisms that influence the TME.

## Abbreviations

CAFs	Cancer-associated Fibroblasts
CRC	Colorectal Cancer
CSFR1	Colony Stimulating Factor 1 Receptor
CTLA-4	Cytotoxic T-lymphocyte-associated Protein 4
CXCL	Chemokine C-X-C Motif Ligand
CXCR	C-X-C Chemokine Receptor
EGFR	Epidermal Growth Factor Receptor
ERBB	Erythroblastic Leukemia Viral Oncogene Homologue Receptors
FAP	Fibroblast Activation Protein
GDP	Guanosine diphosphate
GLI1	GLI family zinc finger 1
GM-CSF	Granulocyye Macrophage Colony-stimulating Factor
GTP	Guanosine triphosphate
Hh	Hedgehog
HIF-1α	Hypoxia Inducible Factor 1 Subunit Alpha
HRAS	Harvey Rat Sarcoma Viral Oncogene Homolog
IGFR	Insulin-like Growth factor
IL	Interleukin
IRF2	Interferon Regulatory factor 2
KRAS	Kirsten Rat Sarcoma Viral Oncogene Homolog
MDSCs	Myeloid-derived Suppressor Cells
MET	Hepatocyte Growth Factor Receptor
MHC I	Major Histocompatibility Class I
MMR	Mismatch Repair
NRAS	Neuroblastoma Rat Sarcoma Viral Oncogene Homolog
NSCLC	Nonsmall Cell Lung Cancer
PD-1	Programmed Cell Death Protein 1
PDAC	Pancreatic Ductal Adenocarcinoma
PD-L1	Programmed Cell Death Protein Ligand 1
RAS	Rat Sarcoma Viral Oncogene Homolog
RTK	Receptor Tirosine Kinase
SHH	Sonic Hedgehog
STING	Stimulator of Interferon Genes
TGFβ1	Transforming Growth Factor Beta 1
TME	Tumor Microenvironment
Tregs	T regulatory cells
